# Autonomic dysfunction in non-critically ill COVID-19 patients during the acute phase of disease: an observational, cross-sectional study

**DOI:** 10.1007/s10072-022-06136-2

**Published:** 2022-05-24

**Authors:** Irene Scala, Simone Bellavia, Marco Luigetti, Valerio Brunetti, Aldobrando Broccolini, Maurizio Gabrielli, Lorenzo Zileri Dal Verme, Paolo Calabresi, Giacomo Della Marca, Giovanni Frisullo

**Affiliations:** 1 Catholic University of Sacred Heart, Rome, Italy; 2 Dipartimento Di Scienze Dell’Invecchiamento, Ortopediche e della Testa-Collo, Fondazione Policlinico Universitario A. Gemelli IRCCS, Neurologiche, Rome, Italy; 3 Department of Emergency, Fondazione Policlinico Universitario A. Gemelli IRCCS, Università Cattolica del Sacro Cuore, Largo Gemelli 8, 00168 Rome, Italy; 4 Digestive Disease Center, Fondazione Policlinico Universitario A. Gemelli IRCCS, Rome, Italy

**Keywords:** SARS-CoV-2, COVID-19, Autonomic dysfunction, Dysautonomia, Orthostatic hypotension, COMPASS-31

## Abstract

**Introduction:**

Evidence is emerging about an extra-pulmonary involvement of SARS-CoV-2, including the nervous system. Autonomic dysfunction in patients recovering from acute coronavirus disease 2019 (COVID-19) has been recently described. Dysautonomic symptoms have been reported in the acute phase of the disease, but clear evidence is lacking, especially in the non-critical forms of the infection.

**Objective:**

The aim of this study is to assess the prevalence of dysautonomia in acute, non-critically ill COVID-19 patients.

**Methods:**

In this observational, cross-sectional study, we compared 38 non-critically ill patients with acute COVID-19 (COVID + group) to 38 healthy volunteers (COVID − group) in order to assess the prevalence of signs and symptoms of dysautonomia through the administration of the composite autonomic symptom score 31 (COMPASS-31) and an active standing test. Comparisons between groups were performed by means of both univariate and multivariate analyses.

**Results:**

The prevalence of orthostatic hypotension was significantly higher in the COVID + group. Higher total scores of COMPASS-31 were observed in the COVID + group than controls. Significant differences between groups emerged in the secretomotor, orthostatic intolerance, and gastrointestinal COMPASS-31 domains. All these results maintained the statistical significance after the adjustment for concomitant drugs with a known effect on the autonomic nervous system assumed by the study participants, except for the differences in the gastrointestinal domain of COMPASS-31.

**Conclusion:**

Our results suggest that an autonomic dysfunction could be an early manifestation of COVID-19, even in the contest of mild forms of the infection.

## Introduction

In December 2019, a novel coronavirus-related disease emerged in Wuhan, China, caused by the severe acute respiratory syndrome coronavirus 2 (SARS-CoV-2). In the following few months, the disease spread through countries and continents, until it was declared pandemic in March 2020.

Although the most common signs and symptoms of SARS-CoV-2 infection are predominantly respiratory, several evidences of extra-pulmonary involvement have been quickly reported, including the involvement of both central and peripheral nervous systems [1–5]. Nonetheless, in the following months, growing evidence raised the suspicion of autonomic nervous system (ANS) involvement, suggested by the occurrence of symptoms such as sweating, postural tachycardia, dizziness, orthostatic intolerance, gastroparesis, constipation, and “mental clouding” [6]. All these dysautonomic symptoms have been extensively described in coronavirus disease 19 (COVID-19) patients even months after the complete cure of SAR-CoV-2 infection [6, 7]. This condition is known as “long COVID”, “long COVID hauler”, or “chronic COVID” [8]. On the other hand, few data are currently available regarding the onset of dysautonomia in the acute phase of SARS-CoV-2 infection, mostly associated to other neurological affection such as acute and subacute polyradiculoneuritis or transverse myelitis [9, 10]. In a previous instrumental study conducted on a similar cohort [11], we found a higher prevalence of pupillary dysfunction, measured by automated pupillometry, and of Sudoscan-detected feet sudomotor dysfunction, in non-critically ill COVID-19 patients than controls.

Moreover, an involvement of the autonomic nervous system has been described in some cases of respiratory infections such as pandemic influenza A (H1N1) [12].

Orthostatic intolerance, the most common presentation of long-COVID-related dysautonomia, includes orthostatic hypotension (OH) due to a deficit of the sympathetic response to orthostatism, resulting in a reduction of peripheral vasoconstriction, peripheral blood accumulation, and consequent reduced cardiac outflow [13]. OH can be classified as neurogenic or non-neurogenic according to the occurrence of the physiological response to the fall in blood pressure, which is particularly based on the compensatory increase in heart rate (HR) [14]. Thanks to its simplicity of use, OH measurement is one of the most common diagnostic tools employed for the assessment of autonomic dysfunction (AD). Recently, the composite autonomic symptom score 31 (COMPASS-31) [15], a self-reported questionnaire initially used in the evaluation of the dysautonomia of diabetic patients, has proven to be a useful tool to investigate the occurrence and severity of AD.

The aim of the current study is to assess the occurrence of AD in non-critically ill COVID-19 patients during the acute phase of disease through the evaluation of OH prevalence and the administration of COMPASS-31.

## Materials and methods

### Study design and population

In this observational single-centre, prospective, cross-sectional study, we included consecutive patients affected by COVID-19 (COVID + group) admitted to the COVID-19 sub-intensive care unit or to the COVID-19 regular ward of IRCCS Fondazione Policlinico Agostino Gemelli, in Rome. Enrolment period went from May 1st, 2021, to December 20th, 2021. The cohort of this study includes all the subjects enrolled in a previous study [11], plus other patients enrolled in the following months.

Inclusion criteria were (1) active SARS-CoV-2 infection at the time of recruitment confirmed by a polymerase chain reaction (PCR) test for SARS-CoV-2; (2) adult age (≥ 18 years); and (3) ability to sign informed consent. Exclusion criteria were as follows: (1) inability to maintain orthostatism for a period of at least 3 min; (2) diabetes; (3) language barrier; (4) continuous positive airway pressure (CPAP) or non-invasive ventilation (NIV); (5) fever at time of evaluation (> 37.5 °C); (6) ongoing infections; (5) cognitive impairment; (6) abnormal neurological examination; and (7) disturbances of state of consciousness.

**Fig. 1:**
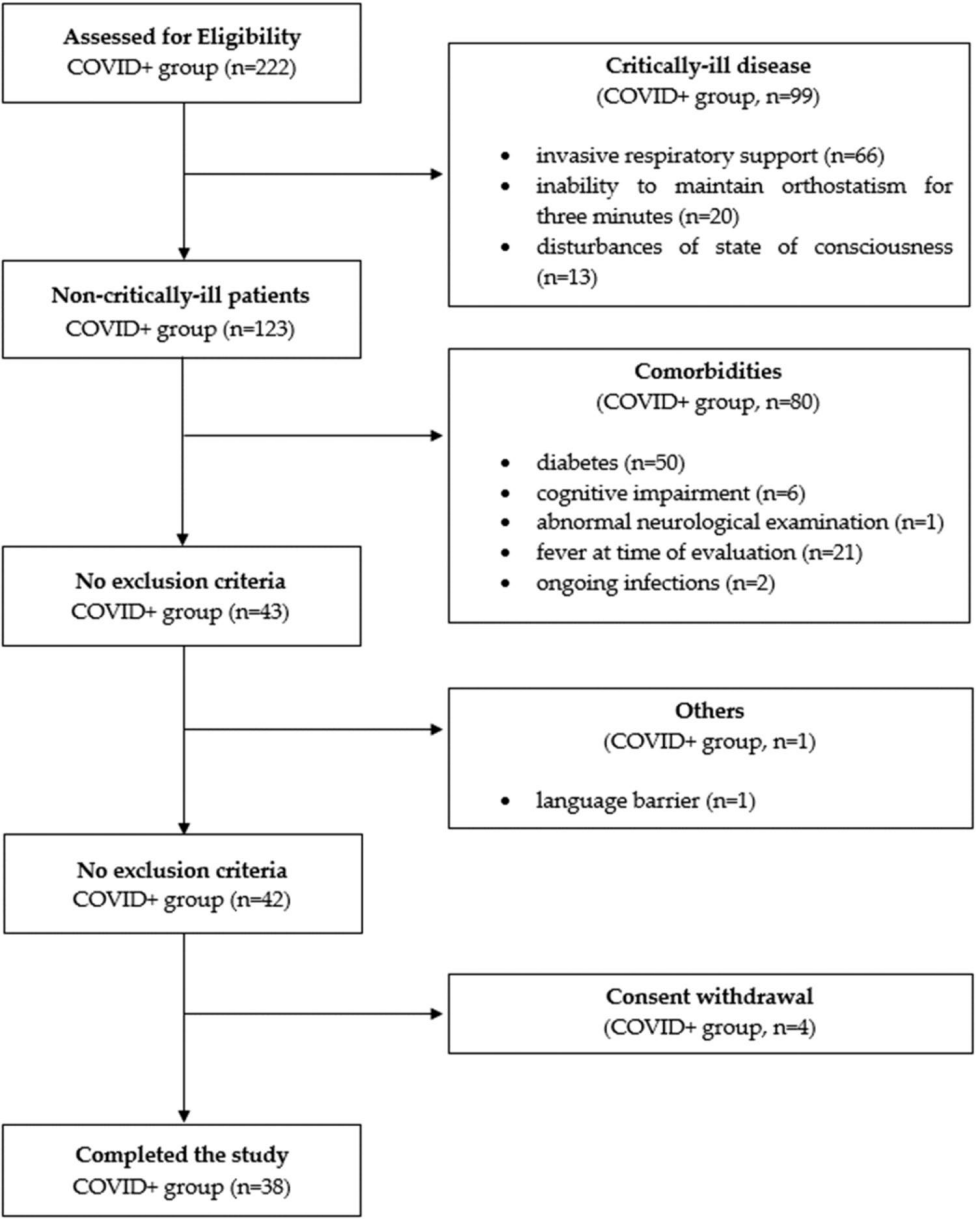
Flow diagram depicting the study enrolment process (COVID + group). Abbreviations: COVID, coronavirus disease 2019

A flow diagram depicting the enrolment process is represented in Fig. 1.

The study complied with the principles of the 1964 Declaration of Helsinki and its later amendments. The research protocol has been approved by the institutional review board — Comitato Etico of Fondazione Policlinico Universitario “A Gemelli” IRCCS – Rome (prot. 0,014,686/21). Written informed consent was obtained from patients and controls at the time of hospital admission.

### Control group

Control group (COVID −) was composed by 38 subjects enrolled among healthy volunteers. 

Inclusion criteria for COVID − group were (1) nasal swab PCR test negative for SARS-CoV-2 infection performed within 48 h; (2) adult age; and (3) ability to sign informed consent. Exclusion criteria were as follows: (1) previous COVID-19; (2) language barrier; (3) abnormal neurological examination; (4) fever; (5) ongoing infections; (6) cognitive impairment or disturbances of state of consciousness.

Each member of the control group was matched with COVID + patients for sex, age, and Body Mass Index (BMI, kg/m^2^). One control subject was enrolled for each case.

### COMPASS-31

COMPASS-31 is a simple and reliable screening tool used to assess the presence and the severity of autonomic symptoms using self-reported measures, first introduced by Sletten et al. in 2012 [14]. The questionnaire is composed by 31 questions concerning six different clinical domains inherent to different functions mediated by the ANS (i.e. orthostatic intolerance, vasomotor, secretomotor, gastrointestinal, bladder, and pupillo-motor). The raw score of every domain is then multiplied with a weight index and the resulting values are added together to obtain the total weighted COMPASS-31 score, which could range from 0 to 100. Higher scores are predictive of more severe autonomic dysfunction [16]. For the present study, a paper format of the Italian validated version of COMPASS-31 [17] was administered to all the members of the COVID + and COVID − groups. All the questions and the optional answers included in the questionnaire were verbally reported to the patients of the COVID + group; therefore, the patients’ answers were written on a paper. COVID − members filled out the questionnaire autonomously. For COVID + group, we asked to answer to the questions referring to the time interval from the onset of COVID-19 symptoms. A score threshold of 17 was established to define the presence of dysautonomia, as previously employed for diabetic autonomic neuropathy [18].

### Active stand test

All the subjects included in the study underwent to an active stand test [19]. Measurements were performed at least 1 h before or after lunch. Arterial blood pressure (BP) was firstly checked after 10 min of supine position through two different measurements separated by 1 min, and again after 3 min of active standing. All the measurements were performed with an automated digital non-invasive BP monitor applied to the left arm. Means of the two lying BP obtained was used in the further analysis. HR monitoring was performed during all the length of measurement (i.e. at least 10 min of supine position followed by 3 min of active standing). Patients admitted to IRCCS Fondazione Policlinico Agostino Gemelli presented an in-room pulse oximeter for oxygen saturation and HR monitoring which were used for HR recording. If not available, such as for control patients, heart rate was assessed by means of a portable pulse oximeter. HR was then registered during the supine position, just before the patients stood up, and after 3 min of active standing.

OH was defined as a sustained reduction of systolic BP of at least 20 mmHg and/or diastolic BP of 10 mmHg within 3 min of standing, as reported by the 2011 Consensus Statement [20]. Since the Consensus Statement suggests that a fall in systolic BP of at least 30 mmHg may be more appropriate for the definition of OH in subjects with supine hypertension [20], we performed a further analysis selecting a cut-off of at least 30 mmHg drop in systolic BP and/or at least 10 mmHg reduction in diastolic BP for subjects who presented a supine BP ≥ 140/90 mmHg.

### Time of evaluation

For COVID + group, the median time between onset of COVID-19 symptoms and time of evaluation was 8 (5–9) days, ranging from 1 to 14 days. COMPASS-31, HR, and OH assessment were performed on the same day for all the study participants.

### Sample size

The calculation of the sample size was carried out considering, on the basis of Romero-Sánchez CM et al. [21], an impairment of autonomic functions in 2.5% of cases. In relation to this hypothesis and, considering a confidence level of 95% and a precision of the estimate of 7%, the minimum number of patients to be included in the study was 19 patients.

### Statistical analysis

All the comparisons were performed between the COVID + and the COVID − group. Normality of data distribution was assessed through the Shapiro–Wilk test. Consequently, normally distributed numerical variables are presented as mean ± standard deviation (SD), while variables presenting a non-normal distribution are expressed as median and interquartile range (IQR). Categorical variables are summarized as number (*n*) and percentage (%). A univariate analysis was initially performed: In order to compare numerical variables, a non-parametric test (Wilcoxon signed-rank test) was used; for categorical variables, we adopted the Fisher’s exact test. Subsequently, to assess the role of possible confounding factors on significant results emerged in the univariate analysis, a multivariate analysis was performed: We adopted a multivariable ordinal logistic regression for numerical variables, while for categorical variables a logistic regression was chosen. Results are reported as adjusted odds ratios (OR) with 95% confidence intervals. Confounding factors were chosen among pharmacological treatments assumed by the study participants based on clinical reasoning: All the drugs with a known effect on autonomic parameters, such as α-blockers, β-blockers, ACE-inhibitors, sartans, calcium channel blockers, antiarrhythmics, and antiepileptic drugs, were included in the multivariate analysis. The Hosmer–Lemeshow test was employed to assess the goodness of fit of the logistic regression model.

The level of significance was set at *p* < 0.05. All statistics were performed by means of the Statistical Package for Social Science (SPSS®) software, version 22 (SPSS®, Inc., Chicago, IL, USA).

## Results

A total of 38 patients in the COVID + group (26/38 males (68.4%); age range 19–86; median age 49.5 (36.5–67.0) years) and 38 healthy volunteers in the COVID − group (26/38 males (68.4%); age range 19–82; median age 51.0 (36.3–61.8) years) were enrolled.

No significant differences were observed between COVID + and COVID − groups in gender (Fisher’s exact test; *p* = 0.597), age (*Z*-test = 364.5, *p* = 0,417), and BMI distribution (COVID + : 24.8 ± 3.3 vs COVID − : 25.6 ± 3.2, *Z*-test = 231.0 *p* = 0.254).

A detailed representation of the demographic and clinical features of the study cohort is available in Table 1.

**Table 1:** Clinical and demographic features of COVID + and COVID − groups. Categorical variables are expressed as number (*n*) and percentage (%). Numerical variables are expressed as median (IQR) or as mean ± SD, as appropriate. Abbreviations: *COVID*, coronavirus disease; *IQR*, interquartile range; *SD*, standard deviation

		COVID + (*n* = 38)	COVID − (*n* = 38)
Clinical features			
Male sex	*n* (%)	26 (68.4%)	26 (68.4%)
Age — years	Median (IQR)	49.5 (36.5–67.0)	51.0 (36.3–61.8)
BMI	Mean ± SD	24.8 ± 3.3	25.6 ± 3.2
COVID symptoms			
Dyspnoea	*n* (%)	18 (47.4%)	0 (%)
Fever at time of evaluation	*n* (%)	0 (0%)	0 (%)
Diarrhoea	*n* (%)	10 (26.3%)	3 (7.9%)
Dizziness	*n* (%)	12 (31.6%)	0 (%)
Pneumonia	*n* (%)	14 (36.8%)	0 (%)
Comorbidity			
Hypertension	*n* (%)	13 (34.2%)	13 (34.2%)
Heart disease	*n* (%)	4 (10.5%)	1 (2.6%)
Dysthiroidism	*n* (%)	4 (10.5%)	3 (7.9%)
Renal failure	*n* (%)	1 (2.6%)	0 (0%)

Concomitant pharmacological treatments of both groups are described in detail in Table 2.

**Table 2:** Concomitant pharmacological treatments of COVID + and COVID − groups. Variables are expressed as number (*n*) and percentage (%). Abbreviations: *COVID*, coronavirus disease

Pharmacological treatments	COVID + (*n* = 38)	COVID − (*n* = 38)
a-blockers	0 (0%)	1 (2.6%)
b-blockers	5 (13.2%)	3 (7.9%)
ACE-inhibitors	4 (10.5%)	5 (13.2%)
Sartans	6 (15.8%)	3 (7.9%)
Calcium channel blockers	3 (7.9%)	2 (5.3%)
Antiarrhythmics	0 (0%)	0 (0%)
Antiepileptic drugs	0 (0%)	1 (2.6%)
Antidepressants	1 (2.6%)	0 (0%)
Antipsychotics	1 (2.6%)	0 (0%)
Hypnotic drugs	2 (5.3%)	3 (7.9%)

### Univariate analysis

For details regarding the results of the univariate analysis, refer to Table 3.

**Table 3:** Results of the univariate analysis. Categorical variables are expressed as number (*n*) and percentage (%), while numerical variables as median (IQR) or as mean ± SD according, respectively, to their not-normal or normal distribution. We can see highlighted the results that reached a statistical significance (*p* < 0.05). Abbreviations: *COVID*, coronavirus disease; *IQR*, interquartile range; *SD*, standard deviation

		COVID + (*n* = 38)	COVID − (*n* = 38)	Wilcoxon signed-rank test		Fisher’s exact test	
				*Z*-test	*p*	*p*	
COMPASS-31							
Total score	Median (IQR)	10 (4–25)	3 (2–8)	154.500	**0.002**		
Domain 1 (orthostatic intolerance)	Median (IQR)	0 (0–12)	0 (0–0)	25.000	**0.014**		
Domain 2 (vasomotor)	Median (IQR)	0 (0–0)	0 (0–0)	6.000	0.083		
Domain 3 (secretomotor)	Median (IQR)	0 (0–6)	0 (0–0)	7.500	** < 0.001**		
Domain 4 (gastrointestinal)	Median (IQR)	4 (1–6)	2 (0–4)	166.000	**0.041**		
Domain 5 (bladder)	Median (IQR)	0 (0–1)	0 (0–0)	43.000	0.550		
Domain 6 (pupillomotor)	Median (IQR)	0 (0–1)	1 (0–1)	217.500	0.285		
Pathologic score (> 17)	*n* (%)	14 (36.8%)	5 (13.2%)			**0.032**	
Orthostatic hypotension test							
Supine position							
Diastolic pressure (mmHg)	Median (IQR)	71 (65–78)	75 (66–80)	375.500	0.717		
Systolic pressure (mmHg)	Mean ± SD	127 ± 17	126 ± 16	335.500	0.803		
Heart rate (bpm)	Median (IQR)	74 (66–80)	68 (62–75)	208.500	0.050		
Standing position							
Diastolic pressure (mmHg)	Mean ± SD	69 ± 12	76 ± 11	506.000	**0.020**		
Systolic pressure (mmHg)	Mean ± SD	118 ± 16	127 ± 15	525.000	**0.025**		
Heart rate (bpm)	Mean ± SD	78 ± 9	69 ± 11	104.500	** < 0.001**		
Δ Diastolic pressure (mmHg)	Median (IQR)	2 (− 3–10)	− 1 (− 8–2)	199000	**0.013**		
Δ Systolic pressure (mmHg)	Median (IQR)	7 (− 1–21)	0 (− 5–5)	120.000	**0.002**		
Δ Heart rate (bpm)	Median (IQR)	3 (− 3–10)	− 1 (− 4–5)	260.500	0.110		
Orthostatic hypotension	*n* (%)	17 (44.7%)	2 (5.3%)			** < 0.001**	
Systolic orthostatic hypotension	*n* (%)	10 (26.3%)	0 (0%)			**0.001**	
Diastolic orthostatic hypotension	*n* (%)	10 (26.3%)	2 (5.3%)			**0.025**	

Bold entries: statistically significance differences (*p* < 0.05)

### COMPASS-31

COMPASS-31 was more frequently above the reference value in COVID + patients than in controls (COVID + : 14/38 (36.8%) vs COVID − 5/38 (13.2%); Fisher’s exact test; *p* = 0.032).

The total weighted COMPASS-31 score showed significantly higher values in the COVID + group than COVID − group (COVID + : 10 (4–25) vs COVID − : 3 (2–8); *Z*-test: 154.500; *p* = 0.002). Analyzing the six clinical domains of COMPASS-31 separately, COVID + patients had higher scores in the orthostatic intolerance (COVID + : 0 (0–12) vs COVID − : 0 (0–0); *Z*-test: 25.000; *p* = 0.014), secretomotor (COVID + : 0 (0–6) vs COVID − : 0 (0–0); *Z*-test: 7.500, *p* < 0.001), and gastrointestinal (COVID + : 4 (1–6) vs COVID − : 2 (0–4); *Z*-test: 166.000; *p* = 0.041) domains. Comparisons among other domains did not show significant differences in the data distributions.

### Active stand test

Concerning the distribution of BP values in clinostatism and orthostatism, COVID + group presented lower orthostatic diastolic (COVID + : 69 ± 12 mmHg vs COVID − : 76 ± 11 mmHg; *Z*-test = 506.000; *p* = 0.020) and systolic (COVID + : 118 ± 16 mmHg vs COVID − : 127 ± 15 mmHg; *Z*-test = 525.000; *p* = 0.025) BP values than COVID − group. The distribution of BP in supine position was similar between two groups. Instead, the BP differences between the two conditions were significantly different between the two groups for both diastolic (COVID + : 2 (− 3–10) mmHg vs COVID − : − 1 (− 8–2) mmHg; *Z*-test = 199.000; *p* = 0.013), and systolic BP values (COVID + : 7 (− 1–21) mmHg vs COVID − : 0 (− 5–5) mmHg; *Z*-test = 120.000; *p* = 0.002).

Adopting the consensus criteria listed above, OH prevalence was significantly higher in COVID + group than in COVID − group (COVID + : 17 (44.7%) vs COVID − : 2 (5.3%); Fisher’s exact test; *p* < 0.001). Within the COVID + group, seven patients presented systolic OH (18.4%), seven patients were diagnosed with diastolic OH (18.4%), while in three patients, we found a significant drop in both the BP components (7.9%). The two COVID − subjects with OH presented a drop in diastolic BP. In the group comparison, both systolic and diastolic OH were significantly more frequent in COVID-19 patients than controls (systolic OH: COVID + : 10 (26.3%) vs COVID − : 0 (0%); Fisher’s exact test; *p* = 0.001 and diastolic OH: COVID + : 10 (26.3%) vs COVID − : 2 (5.3%); Fisher’s exact test; *p* = 0.025).

Supine hypertension at the time of the active stand test was similarly represented between groups (COVID + : 11 (28.9%) vs COVID − : 12 (31.6%); Fisher’s exact test; *p* = 1.000). Considering the aforementioned cut-off for subjects with supine hypertension (i.e. a reduction of 30 mmHg in the systolic BP and/or a 10 mmHg drop in the diastolic BP), only one COVID + patient previously diagnosed with systolic OH no longer presented this condition, while for the COVID − group, no change was detected. Also in this analysis, COVID + subjects presented a much higher prevalence of OH than controls (COVID + : 16 (42.1%) vs COVID − : 2 (5.3%); Fisher’s exact test; *p* < 0.001).

Concerning the HR analysis, the distribution of orthostatic HR differed between groups (COVID + : 78 ± 9 beats per minute (bpm) vs COVID − : 69 ± 11 bpm; *Z*-test = 104.500; *p* < 0.001), while the distribution of supine HR and of HR differences between the two aforementioned conditions was similar between COVID-19 patients and controls.

### Multivariate analysis

A summary of the results of the multivariate analysis is available in Table 4.

**Table 4:** A table depicting the results of the multivariate comparisons between COVID + and COVID − group, after the adjustment for concomitant drugs assumed by the study participants. Abbreviations: *CI*, confidence interval

	Effect variable	Adjusted value (95%CI)	*p*
Orthostatic hypotension test			
Orthostatic hypotension	Odds ratio	16.00 (3.22–79.48)	***p* = 0.001**
Diastolic pressure (mmHg)	Beta	− 6.66 (− 11.98 to − 1.33)	***p* = 0.015**
Systolic pressure (mmHg)	Beta	− 7.52 (− 14.62 to − 0.420)	***p* = 0.038**
COMPASS31			
Total score	Beta	7.86 (2.60–13.12)	***p* = 0.004**
Domain 1 (orthostatic intolerance)	Beta	4.42 (0.85–8.00)	***p* = 0.016**
Domain 3 (secretomotor)	Beta	2.32 (1.03–3.61)	***p* = 0.001**
Domain 4 (gastrointestinal)	Beta	1.34 (− 0.07–2.74)	*p* = 0.061
Pathologic score (> 17)	Odds ratio	3.60 (1.10–11.85)	***p* = 0.035**

Bold entries: statistically significance differences (*p* < 0.05)

### COMPASS-31

After the adjustment for aforementioned pharmacological treatments, we confirmed that global COMPASS-31 scores were significantly higher in COVID-19 patients than controls (Beta = 7.86; CI = 2.60–13.12; *p* = 0.004). Moreover, COVID-19 patients presented more frequently pathologic values of COMPASS-31 (OR 3.6; CI = 1.10–11.85; *p* = 0.035) than COVID − group also in the multivariate analysis. Analyzing the single COMPASS-31 domains that had resulted significantly different between groups in the univariate analysis, we found, in COVID + patients, higher scores in the orthostatic intolerance (Beta = 4.42; CI = 0.85–8.00; *p* = 0.016), and in the secretomotor (Beta = 2.32; CI = 1.03–3.61; *p* = 0.001) domains, but not in the gastrointestinal (Beta = 1.34; CI =  − 0.07–2.74; *p* = 0.061) one in the multivariable ordinal logistic regression.

### Active stand test

Also in the logistic regression, after the adjustment for the previously specified variables, COVID-19 patients showed higher OH prevalence (OR 16.00; CI = 3.22–79.48; *p* = 0.001) than controls. Moreover, in the multivariate ordinal logistic regression, COVID-19 patients continued to present significantly lower values of both systolic (Beta =  − 7.52; CI =  − 14.62 to − 0.420; *p* = 0.038) and diastolic (Beta =  − 6.66; CI =  − 11.98 to − 1.33; *p* = 0.015) orthostatic BP than COVID − group.

## Discussion

In this study, we found a higher prevalence of clinical signs and symptoms of dysautonomia in COVID-19 patients than controls. In particular, OH was observed almost exclusively in the COVID + group. Even more, COMPASS-31 showed a higher frequency of dysautonomic symptoms in COVID + group than COVID − subjects.

The occurrence of AD is increasingly reported as a clinical feature of long-COVID syndrome [22]. On the other hand, evidence of AD after the acute phase of other coronavirus infection, such as SARS, has already been reported in last years [23]. OH is a simply assessed and clinical evident condition attributable, in most cases, to a sympathetic dysfunction leading to a lack of peripheral vasoconstriction [13]. The reduction of the cardiac outflow can result, when the drop in BP overcomes the autoregulatory capacities of the cerebral and retinal circulations, in symptoms of systemic hypoperfusion, such as fatigue, altered mentation, dizziness, light-headedness, blurred vision, or even syncope [24]. These AD symptoms have been frequently reported as major clinical features of long-COVID syndrome [6, 7], but poor data have been collected during the acute phase of SARS-CoV-2 infection. In particular, Aragòn-Benedì et al. [25], in a heart rate variability study conducted on 14 critically ill COVID-19 patients, reported a misalignment of the sympathetic/parasympathetic balance with a slight predominance of the parasympathetic system; consequently, sympathetic depletion was associated with worse outcome. In relation to the high disease severity of the patients included in their study, the authors speculated that autonomic dysregulation likely represented the cause and effect of the different stages of SARS-CoV-2 disease, the severe inflammatory system response syndrome, and its compensatory anti-inflammatory response. However, this hypothesis does not appear to be confirmed by our study, as AD was also observed in patients with a mild form of COVID-19, with no evidence of clinical signs of inflammation, suggesting a different pathogenetic mechanism of dysautonomia. Eshak et al. [26] described the case of a patient admitted to intensive care unit with a wide range of BP fluctuations throughout the day; in consideration of the negativity of blood culture, labile BP was interpreted as an AD manifestation. Moreover, Suresh et al. [27] reported a case of an acute COVID-19 patient with severe OH which improved with midodrine and fludrocortisone, suggesting the dysautonomic aetiology.

Regarding the aetiology of OH in our sample, the clinical evaluation performed on this study does not allow to find an answer about the possible neurogenic nature of OH. However, even if HR values collected during the orthostatic position were higher in the COVID + group than controls, HR did not significantly increase in the COVID + group despite an evident drop in systolic and/or diastolic blood pressure. This finding could suggest a neurogenic nature of SARS-CoV-2-related OH [28].

COMPASS-31, a screening tool for dysautonomia mostly employed to evaluate the occurrence of small fibres neuropathies in diabetes [29], was recently adopted in other neurological conditions, such as multiple sclerosis and parkinsonism [30, 31]. Adopting this dysautonomic tool, we observed in our sample a prevalence of AD of 36.8%, defined as a total COMPASS-31 score > 17. Moreover, a significant, self-reported, impairment of secretomotor, and orthostatic intolerance domains was observed in COVID + group. The accordance between the self-reported autonomic symptoms, in particular regarding the orthostatic intolerance domain, and the objective finding of OH in our study sample, suggests the effectiveness of COMPASS-31 in detecting AD. This data is further strengthened by the finding, in a previous study [11], of sudomotor dysfunction in a similar cohort of non-critically ill COVID-19 patients.

In the framework of COVID-19-related dysautonomia, COMPASS-31 has been already adopted in two previous studies, only concerning long-COVID patients [32, 33]. Anaya et al. [32] administered the questionnaire to 100 patients affected by long-COVID and then clustered the population in two groups basing on COMPASS-31 scores. One cluster exhibited higher scores of COMPASS-31 than the other, accounting for 31% of the population. Buoite Stella et al. [33] found a median COMPASS-31 score of 17.6 in 108 participants with previous SARS-CoV-2 infection with a prevalent involvement of orthostatic intolerance, pupillomotor, sudomotor, and gastrointestinal domains. Our results are in line with previous reports.

The results of this study, in accordance with our previous instrumental findings of a complex misalignment of the ANS in a similar cohort [11], suggest that AD could be one of the clinical features of COVID-19, also in non-critically ill patients.

This is the first study reporting a correlation between acute, non-critical COVID-19, and clinical AD, assessed by objective and subjective tools. Moreover, we found an accordance between COMPASS-31 dysautonomic symptoms and objective signs of dysautonomia. The principal limitation of our study is the small sample size. We should also consider that the concomitant comorbidities of study participants and the sub-intensive care unit setting in which some patients of the COVID + group were admitted could alter the autonomic functioning. Moreover, we adopted a dysautonomic questionnaire mostly employed to evaluate chronic diseases to an acute condition, modifying the reference time intervals. Finally, the study was not blinded and consequently both the assessor evaluation and the patients reporting could be altered by a reporting bias.

## Conclusions

According to the results of the present study and to growing clinical evidence, dysautonomia seems to play an important role among the clinical manifestations in both the acute and chronic phase of SARS-CoV-2 infection. However, due to the small sample size, further blinded studies performed on a larger cohort of patients may be useful to confirm these data and to trace the specific pathogenic mechanism of autonomic dysfunctions.

## Data Availability

The datasets generated during and/or analysed during the current study are available from the corresponding author on reasonable request.
